# Ciliary Neurotrophic Factor Acts on Distinctive Hypothalamic Arcuate Neurons and Promotes Leptin Entry Into and Action on the Mouse Hypothalamus

**DOI:** 10.3389/fncel.2020.00140

**Published:** 2020-05-21

**Authors:** Wiebe Venema, Ilenia Severi, Jessica Perugini, Eleonora Di Mercurio, Marco Mainardi, Margherita Maffei, Saverio Cinti, Antonio Giordano

**Affiliations:** ^1^Section of Neuroscience and Cell Biology, Department of Experimental and Clinical Medicine, Università Politecnica Delle Marche, Ancona, Italy; ^2^Institute of Neuroscience, National Research Council, Pisa, Italy; ^3^Center of Obesity, Università Politecnica delle Marche-United Hospitals, Ancona, Italy

**Keywords:** arcuate nucleus, AgRP neurons, NPY neurons, tanycytes, median eminence, obesity, leptin resistance, STAT3

## Abstract

In humans and experimental animals, the administration of ciliary neurotrophic factor (CNTF) reduces food intake and body weight. To gain further insights into the mechanism(s) underlying its satiety effect, we: (i) evaluated the CNTF-dependent activation of the Janus kinase 2 (JAK2) and signal transducer and activator of transcription 3 (STAT3) pathway in mouse models where neuropeptide Y (NPY) and pro-opiomelanocortin (POMC) neurons can be identified by green fluorescent protein (GFP); and (ii) assessed whether CNTF promotes leptin signaling in hypothalamic feeding centers. Immunohistochemical experiments enabled us to establish that intraperitoneal injection of mouse recombinant CNTF activated the JAK2-STAT3 pathway in a substantial proportion of arcuate nucleus (ARC) NPY neurons (18.68% ± 0.60 in 24-h fasted mice and 25.50% ± 1.17 in fed mice) but exerted a limited effect on POMC neurons (4.15% ± 0.33 in 24-h fasted mice and 2.84% ± 0.45 in fed mice). CNTF-responsive NPY neurons resided in the ventromedial ARC, facing the median eminence (ME), and were surrounded by albumin immunoreactivity, suggesting that they are located outside the blood-brain barrier (BBB). In both normally fed and high-fat diet (HFD) obese animals, CNTF activated extracellular signal-regulated kinase signaling in ME β1- and β2-tanycytes, an effect that has been linked to the promotion of leptin entry into the brain. Accordingly, compared to the animals treated with leptin, mice treated with leptin/CNTF showed: (i) a significantly greater leptin content in hypothalamic protein extracts; (ii) a significant increase in phospho-STAT3 (P-STAT3)-positive neurons in the ARC and the ventromedial hypothalamic nucleus of normally fed mice; and (iii) a significantly increased number of P-STAT3-positive neurons in the ARC and dorsomedial hypothalamic nucleus of HFD obese mice. Collectively, these data suggest that exogenously administered CNTF reduces food intake by exerting a leptin-like action on distinctive NPY ARC neurons and by promoting leptin signaling in hypothalamic feeding centers.

## Introduction

In mammals, the energy balance and body weight are regulated by complex, redundant and overlapping brain neural circuits which largely operate in response to circulating metabolic hormones such as insulin, leptin, and ghrelin (Schwartz et al., 2000; Waterson and Horvath, 2015; Clemmensen et al., 2017; Timper and Brüning, 2017). Over the past few decades leptin, the main “satiety” factor, has been the paradigm for the action of the metabolic hormones on the hypothalamic centers that control feeding and the energy balance (Zhang et al., 1994; Friedman, 2016). Leptin is secreted into the bloodstream from white adipocytes in proportion to the body fat stores. In normal conditions it reaches the hypothalamic parenchyma, where its interaction with neurons bearing the functional leptin receptor (LepRb) reduces food search and intake and promotes short- and long-term energy-intensive behaviors like thermogenesis, locomotor activity, and reproduction. Activation of the Janus kinase 2 (JAK2) and signal transducer and activator of transcription 3 (STAT3) pathway in these hypothalamic neurons is essential for leptin to exert its full metabolic effect, as demonstrated by the hyperphagic and obese phenotypes found in mice where LepRb mutations at tyrosine 1138 prevent STAT3 activation (Bates et al., 2003) and in those lacking STAT3 in LepRb-bearing neurons (Piper et al., 2008). In experimental animals, immunohistochemical evaluation of nuclear phospho-STAT3 (P-STAT3) expression after administration of an intraperitoneal or intravenous bolus of leptin is a useful and sensitive method to assess the hormone’s action in the hypothalamus. By this technique, the leptin blood surge induces strong P-STAT3 immunoreactivity in several neurons of the arcuate nucleus (ARC), ventromedial nucleus (VMH), dorsomedial nucleus (DMH) and perifornical-lateral hypothalamic area (PF-LHA; Hübschle et al., 2001; Münzberg et al., 2003; Frontini et al., 2008). In physiological conditions, circulating leptin is held to induce first of all strong JAK2-STAT3 activation in the neurons of the ARC, which is characterized by considerable vascular permeability (Mullier et al., 2010; Morita and Miyata, 2012); signaling subsequently propagates to second-order neurons, which are found in more dorsal, metabolically relevant nuclei, where the hypothalamic parenchyma is protected from circulating substances by a tightly sealed blood-brain barrier (BBB; Obermeier et al., 2013). Leptin’s action in the ARC—where it inhibits orexigenic agouti-related peptide and neuropeptide Y (NPY)-containing neurons and excites anorexigenic satiety-promoting cocaine- and amphetamine-regulated transcripts and pro-opiomelanocortin-containing (POMC) neurons—is critical for its satiety effect (Schwartz et al., 2000; Timper and Brüning, 2017).

Ciliary neurotrophic factor (CNTF) has been discovered in extracts of chick intraocular tissue, where it promoted the survival of ciliary ganglion neurons (Adler et al., 1979). It was subsequently purified and cloned also in mammals where, in addition to other trophic actions, it was found to support the survival of brain motor neurons (Sendtner et al., 1994). These observations led human recombinant CNTF to be tested in amyotrophic lateral sclerosis patients where, although it failed to slow disease progression, it unexpectedly induced anorexia and weight loss (ACTS, 1996; Miller et al., 1996). Importantly, the weight-reducing effect of exogenous CNTF was also observed in leptin-resistant obese patients treated with subcutaneous Axokine, a human CNTF with enhanced specificity and potency (Ettinger et al., 2003). These findings have raised considerable interest in the metabolic role of CNTF and have suggested the possibility of using this peptide, or its analogs, to treat human obesity and associated diseases (Findeisen et al., 2019). In the past few years, studies of animal models have suggested two putative mechanisms through which CNTF may induce the anorectic response: activation of JAK2-STAT3 signaling in hypothalamic ARC NPY and POMC neurons (Lambert et al., 2001; Anderson et al., 2003; Janoschek et al., 2006), thus exerting a leptin-like action, and generation over time of new leptin-responsive neurons in the mediobasal hypothalamus (Kokoeva et al., 2005), thus amplifying the responsiveness to circulating leptin of the hypothalamic centers controlling feeding and the energy balance. Subsequent studies have however challenged both hypotheses, since transgenic mice with a conditional deletion of CNTF receptor α (CNTFRα, a CNTFR subunit) in LepRb-bearing neurons displayed no change in the anorectic response to Axokine (Stefater et al., 2012), suggesting that CNTFR signaling in leptin-responsive neurons is not required for the CNTF-induced satiety effect; moreover, intracerebral Axokine infusion for a week did produce a satiety effect in high-fat-fed obese mice, but not substantial neurogenesis in the mediobasal hypothalamus (Borg et al., 2016).

We have previously documented that in the hypothalamus CNTF—besides activating STAT3 in ARC neurons—also induces STAT3, STAT1, and STAT5 phosphorylation in median eminence (ME) cells and that a considerable proportion of CNTF-responsive ME cells were ependymal and glial cells displaying markers of immaturity (Severi et al., 2012, 2015; Senzacqua et al., 2016). These findings suggest that the anorectic effect of CNTF may also involve the ME, the circumventricular organ of the tuberal hypothalamus which plays a role in the access of circulating satiety factors to the hypothalamic nuclei involved in energy balance regulation (Langlet et al., 2013b; Obermeier et al., 2013; Morita-Takemura and Wanaka, 2019). To gain further insights into the mechanisms underlying the satiety-inducing action of CNTF, we evaluated the CNTF-dependent activation of the JAK2-STAT3 pathway in mouse models where NPY and POMC neurons can be identified by a green fluorescent protein (GFP) then tested whether the satiety effect induced by CNTF could be due, at least partly, to enhanced leptin entry into and action on the hypothalamic feeding centers.

## Materials and Methods

### Animals, Treatments and Tissue Processing

Wildtype and *db*/*db* and *db*/*+* C57/BL6 mice were purchased from Charles River (Lecco, Italy). Mice expressing GFP under the *NPY* (B6.FVB-Tg(Npy-hrGFP)1Lowl/J, stock #006417) and the *POMC* [C57BL/6J-Tg(Pomc-EGFP)1Lowl/J, stock #009593] promoter, obtained from Jackson Laboratories (Bar Harbor, ME, USA), were maintained on a C57/BL6 background. Mice were housed individually in plastic cages under constant environmental conditions (12 h light/dark cycle at 22°C) with *ad libitum* access to food and water. Animal care was according to Council Directive 2010/63/UE and all experiments were approved by the Italian Health Ministry (authorization no. 405/2018-PR). All mice initially received a standard low-fat diet (Charles River; 19 kJ% from fat, 50 kJ% from carbohydrates and 31 kJ% from proteins); when they were 4 weeks old, some wildtype mice were switched to a high-fat diet (HFD; Charles River; 50 kJ% from fat, 30 kJ% from carbohydrates and 20 kJ% from proteins) for 10 weeks. All experiments were carried out in 12- to 14-week-old male mice. Before sacrifice, some mice were treated for 45 min with intraperitoneal recombinant rat CNTF (0.3 mg/kg of body weight; R&D Systems, Minneapolis, MN, USA); with mouse recombinant leptin (0.3 mg/kg of body weight; Sigma-Aldrich, St Louis, MO, USA), with both CNTF and leptin; or with the same volume of vehicle. For Western blotting, anesthetized animals were decapitated, the brain was rapidly removed from the skull and placed with its ventral side up in a pre-cooled adult mouse coronal brain matrix (ASI Instruments, Warren, MI, USA); the hypothalamus was dissected out, immediately snap-frozen in liquid nitrogen and stored at −80°C. Histological examination confirmed that these specimens contained the whole hypothalamus, from the preoptic area to the mammillary body, including the entire third ventricle. For morphological studies, anesthetized animals were perfused transcardially with 4% paraformaldehyde in 0.1 M phosphate buffer (PB), pH 7.4. Brains were carefully removed from the skull, postfixed with the same fixative solution for 24 h at 4°C and washed in PB. Free-floating coronal sections (40 μm thick) were obtained with a Leica VT1200S vibratome (Leica Microsystems, Vienna, Austria) and kept in phosphate-buffered saline (PBS), pH 7.4, at 4°C until use.

### Antibodies

The primary and secondary antibodies used in the study are reported in Supplementary Table S1.

### Western Blotting

Proteins were extracted using a lysis buffer containing 50 mM Tris-HCl (pH 7.4), 1% NP-40, 1 mM EDTA, 150 mM NaCl, 1 mM sodium orthovanadate, 0.5% sodium deoxycholate, 0.1% sodium dodecyl sulfate (SDS), 2 mM phenylmethylsulfonylfluoride and 50 mg/ml aprotinin. Tissue fragments were homogenized by passing them through a 26-gauge needle and sonicated. Lysates were cleared by centrifugation at 14,000 rpm for 20 min at 4°C and protein concentrations were determined by the Bradford assay (Bio-Rad Laboratories, Segrate, Italy). Proteins were size-fractionated in precast 4–12% Bis-Tris polyacrylamide gels (NW04120, Invitrogen, Carlsbad, CA, USA) by SDS-PAGE. After electrophoresis, they were transferred to a nitrocellulose membrane using Bio-Rad’s Trans-Blot TurboTM Transfer system. Loading and transfer efficiency were assessed by Ponceau S membrane staining (Santa Cruz Biotechnology, Santa Cruz, CA, USA). Membranes were then blocked for 1 h in TBS-Tween-20 [50 mM Tris-HCL (pH 7.6), 200 mM NaCl and 0.1% Tween-20] containing 5% non-fat dried milk and incubated with the primary antibody overnight at 4°C (Supplementary Table S1). To visualize the immunoreaction, the blots were incubated with horseradish peroxidase-conjugated secondary antibodies (Supplementary Table S1) for 1 h; the bands were visualized with the Chemidoc Imaging system using the Clarity™ Western ECL chemiluminescent substrate (all from Bio-Rad). The immunoreactive bands were quantitated with Bio-Rad’s Image Lab software. Where appropriate, membranes were stripped, washed, and re-probed for total protein content.

### Immunohistochemistry, Confocal Microscopy, and Morphometric Analysis

Unmasking procedures were used for immunohistochemical experiments (Frontini et al., 2008). Free-floating sections were reacted with 1% NaOH and 1% H_2_O_2_ (20 min), 0.3% glycine (10 min) and 0.03% SDS (10 min). After rinsing in PBS, they were blocked with 3% normal goat serum (in 0.2% Triton X-100; 60 min) and incubated with the primary antibody in PBS (Supplementary Table S1), overnight at 4°C. After a thorough rinse in PBS, sections were incubated in 1:200 v/v biotinylated secondary antibody solution (in PBS; 30 min; Supplementary Table S1), rinsed in PBS and incubated in the avidin-biotin-peroxidase complex (ABC Elite PK6100, Vector), washed several times in PBS and finally incubated in 3,3′-diaminobenzidine tetrahydrochloride (0.05% in 0.05 M Tris with 0.03% H_2_O_2_; 5 min). After immunohistochemical staining, sections were mounted on slides, air-dried, dehydrated in ethanol, cleared with xylene, and covered with Entellan. Staining was never observed when the primary antibody was omitted. P-STAT3-positive neurons were counted on each side in 3 alternate coronal sections of the tuberal part of the hypothalamus of mice treated with leptin and leptin/CNTF, then left and right values were averaged for each slice. Three mice per group were analyzed. The location of individual nuclei and areas was precisely identified by Nissl staining in adjacent sections according to Paxinos and Franklin (2001).

For immunofluorescence and multiple-labeling experiments, free-floating sections were processed as described above up to and including incubation with the primary antibodies. The section was then incubated overnight in a mixture of two or three primary antibodies (Supplementary Table S1). The next day sections were washed twice with PBS and incubated in a cocktail of fluorophore-linked secondary antibodies at a dilution of 1:100 in PBS for 1 h at room temperature (Supplementary Table S1). Sections were subsequently washed twice with PBS, stained with TO-PRO3, mounted on standard glass slides, air-dried, and coverslipped using Vectashield mounting medium (Vector). Sections were viewed under a motorized Leica DM6000 microscope at different magnifications. Fluorescence was detected with a Leica TCS-SL spectral confocal microscope equipped with an Argon and He/Ne mixed gas laser. Fluorophores were excited with the 488 nm, 543 nm, and 649 nm lines and imaged separately. Images (1,024 × 1,024 pixels) were obtained sequentially from two channels using a confocal pinhole of 1.1200 and stored as TIFF files. The brightness and contrast of the final images were adjusted using Photoshop 6 (Adobe Systems, Mountain View, CA, USA). The percentage of GFP-positive neurons co-expressing P-STAT3 and the percentage of P-STAT3-positive nuclei contained into GFP-expressing NPY or POMC neurons were calculated in three coronal sections of the hypothalamic ARC that were representative of its rostral (Bregma from −1.46 to −1.70 mm), intermediate (Bregma from −1.70 to −1.94 mm) and caudal (Bregma from −1.94 to 2.18 mm) subdivisions according to Paxinos and Franklin (2001). In GFP-expressing NPY mice, GFP-positive cells were visualized using direct fluorescence, whereas in GFP-expressing POMC mice they were visualized by GFP immunohistochemistry. Three mice per group were analyzed. In each section, both sides were evaluated at 40× magnification and examined on a fixed confocal plane. For the evaluation of the percentage of GFP-positive neurons co-expressing P-STAT3, only GFP-positive cells showing a corresponding TO-PRO3-stained nucleus were included in the counts. About 500 and 300 GFP-positive ARC neurons per mouse were examined in GFP-NPY and GFP-POMC mice, respectively. For the evaluation of the percentage of P-STAT3-positive nuclei contained into GFP-expressing NPY or POMC neurons, neuron-like P-STAT3-positive nuclei (large, roundish and provided with nucleoli) were evaluated. About 30 and 100 nuclei per mouse were examined in vehicle-injected and CNTF-treated 24-h fasted mice, respectively; about 350 and 150 nuclei per mouse were examined in vehicle-injected and CNTF-treated normally fed mice, respectively.

### Statistical Analysis

All values are mean ± standard error of the mean (SEM). Data were analyzed for significance with GraphPad Prism (version 8) using one-way ANOVA. Differences between groups were analyzed using an unpaired student’s *t*-test. The threshold for significance was set at *p* < 0.05.

## Results

### CNTF Predominantly Activates the JAK2-STAT3 Pathway in ARC NPY Neurons of 24 h Fasted Mice

**Figure 1:**
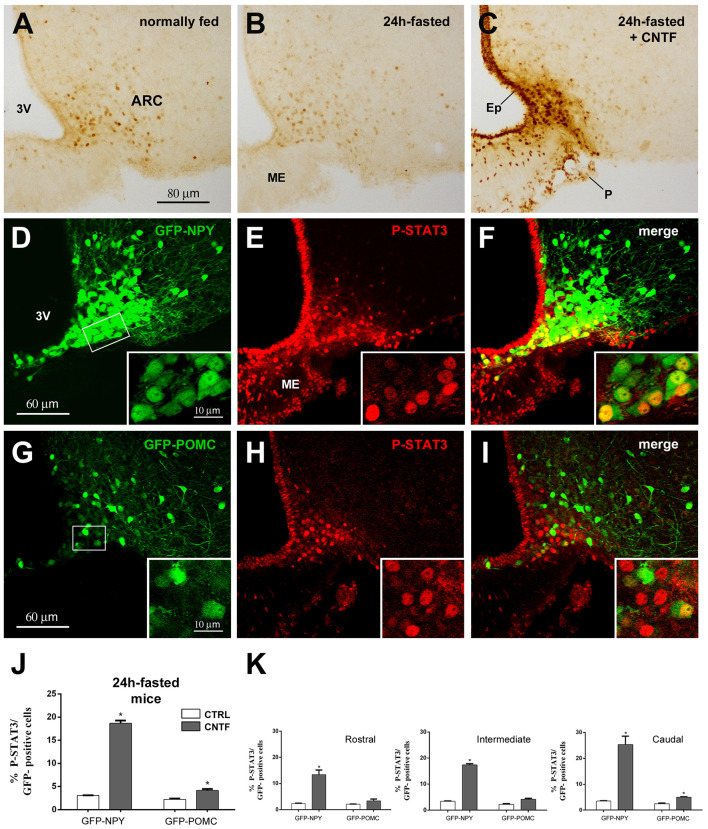
Janus kinase 2 (JAK2)-signal transducer and activator of transcription 3 (STAT3) pathway activation by ciliary neurotrophic factor (CNTF) in hypothalamic ARC neurons. **(A–C)** Immunoperoxidase staining showing phospho-STAT3 (P-STAT3) immunoreactivity in a normally fed mouse **(A)**, in a 24-h fasted mouse **(B)**, and a CNTF-treated 24-h fasted mouse **(C)**. **(D–F)** Representative confocal images from a CNTF-treated green fluorescent protein (GFP)-expressing neuropeptide Y (NPY) mouse showing some NPY neurons (**D**, green) in the ventromedial part of the ARC also expressing P-STAT3 (**E**, red). **(G–I)** Representative confocal images from a CNTF-treated GFP-expressing pro-opiomelanocortin (POMC) mouse showing a few POMC neurons (**G**, green) in the ventromedial part of the ARC also expressing P-STAT3 (**H**, red). ARC, arcuate nucleus; ME, median eminence; 3V third ventricle; Ep, ependymal layer; P, pial surface. Insets are enlargement of the framed areas in **(D,G)**. **(J,K)** Percentage of GFP-expressing NPY and POMC neurons that are also positive for P-STAT3 in the whole ARC **(J)** and its rostral (Bregma from −1.46 to −1.70 mm), intermediate (Bregma from −1.70 to −1.94 mm) and caudal (Bregma from −1.94 to 2.18 mm) subdivisions **(K)** from vehicle-injected (CTRL) and CNTF-treated (CNTF) 24-h fasted mice. All pictures were taken from coronal hypothalamic sections at Bregma −1.70 mm. Data (*n* = 3) are mean ± standard error of the mean (SEM), *p < 0.05 compared to control mice (unpaired student’s *t*-test).

In hypothalamic ARC neurons, immunohistochemical evaluation of the activation of JAK2-STAT3 signaling after treatment with any metabolic hormone is hampered by a certain amount of P-STAT3 immunoreactivity, which is found in normally fed mice as a result of endogenous circulating leptin (Figure 1A; Faouzi et al., 2007; Frontini et al., 2008). Fasting for 24 h reduces circulating leptin as well as basal P-STAT3 immunoreactivity (Figure 1B). Thus, to establish whether and to what extent CNTF induced JAK2-STAT3 activation in ARC neurons, mouse recombinant CNTF was administered after 24-h fasting and P-STAT3 immunoreactivity was evaluated in coronal sections of the hypothalamus. As found in previous studies, CNTF induced strong STAT3 activation in the ependyma lining the third ventricle, in scattered ME cells and neuron-like ARC cells (Lambert et al., 2001; Anderson et al., 2003; Janoschek et al., 2006; Severi et al., 2012, 2015); notably, however, STAT3 activation in the ARC was confined to its ventromedial portion (Figure 1C). The phenotype of the ARC neurons targeted by CNTF was determined using GFP-expressing NPY and GFP-expressing POMC mice. In GFP-expressing NPY mice fasted for 24 h, CNTF injection-induced P-STAT3 nuclear staining in GFP-positive neurons of the ventromedial ARC (Figures 1D–F). Rare GFP-positive neurons scattered in the ME also displayed P-STAT3-positive nuclei. In GFP-expressing POMC mice fasted for 24 h, CNTF resulted in the presence of very few GFP- and P-STAT3-positive neurons. Interestingly, the POMC neurons targeted by CNTF were again located in the most ventromedial part of the ARC (Figures 1G–I). Quantitative analysis revealed that, whereas in vehicle-injected fasted mice STAT3 phosphorylation was detectable in as few as 3.06% ± 0.05 of GFP-expressing NPY neurons and 2.23% ± 0.19 of GFP-expressing POMC neurons, CNTF treatment-induced P-STAT3 immunoreactivity in 18.68% ± 0.60 of GFP-expressing NPY neurons and in 4.15% ± 0.33 of GFP-expressing POMC neurons (Figure 1J). To assess whether CNTF-responsive neurons exhibited a preferential distribution along the rostral-to-caudal axis, the ARC was divided into three regions: rostral (Bregma from −1.46 to −1.70 mm), intermediate (Bregma from −1.70 to −1.94 mm) and caudal (Bregma from −1.94 to 2.18 mm). After CNTF treatment the number of P-STAT3-positive NPY neurons progressively increased from the rostral to the caudal region, whereas the few P-STAT3-positive POMC neurons were preferentially located in the caudal region of the ARC (Figure 1K). Besides NPY and POMC neurons, other classes of neuronal cells reside in the hypothalamic ARC-ME complex (Campbell et al., 2017). To obtain insights on non-NPY and non-POMC neuronal cells eventually activated by CNTF, we counted how many P-STAT3-positive neuronal nuclei were contained into NPY or POMC neurons. Results showed that in vehicle-injected animals 81.53% ± 0.99 of P-STAT3-positive nuclei were located into NPY neurons and 21.73% ± 0.63 of them were located into POMC neurons; in CNTF-treated mice, 78.40% ± 0.55 of P-STAT3-positive nuclei were into NPY neurons and 22.87% ± 0.99 of them into POMC neurons. Even whether our analysis was limited by that P-STAT3-positive neurons were merely distinguished through the morphology of their nucleus (large, roundish, and provided with nucleoli), these data suggest that under fasting conditions administered CNTF exclusively acts on NPY and POMC ARC neurons.

### CNTF Predominantly Activates the JAK2-STAT3 Pathway in ARC NPY Neurons of Normally Fed Mice

**Figure 2:**
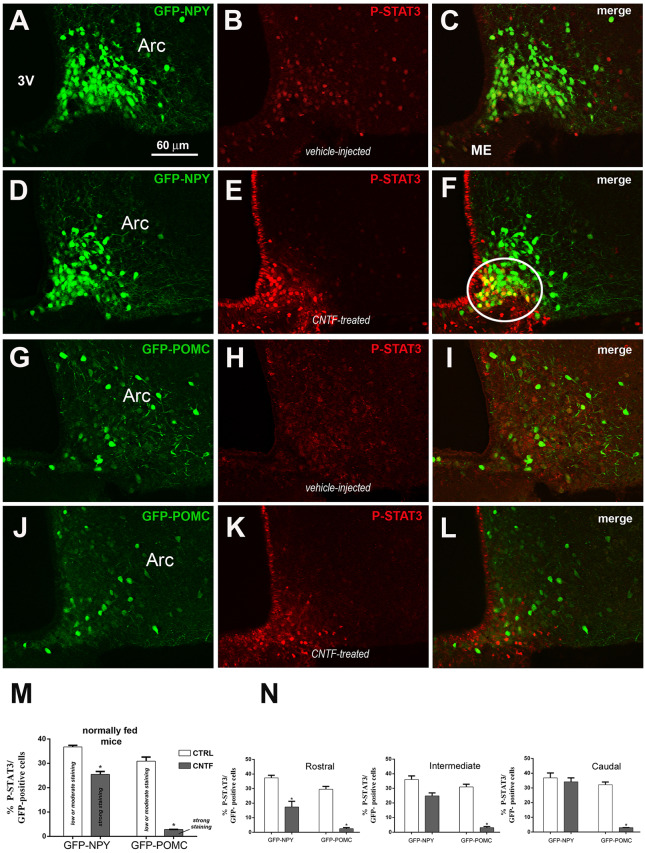
JAK2-STAT3 pathway activation by CNTF in hypothalamic ARC neurons of normally fed mice. **(A–F)** Representative confocal images from vehicle-injected **(A–C)** and CNTF-treated **(D–F)** GFP-expressing NPY mice. **(G–L)** Representative confocal images from vehicle-injected **(G–I)** and CNTF-treated **(J–L)** GFP-expressing POMC mice. In both mouse models, vehicle-injected mice exhibit low to moderate P-STAT3 staining in several neurons which are uniformly distributed in the ARC **(B,H)**; CNTF treatment led to a reduction in the number of P-STAT3-positive neurons which are located in the most ventromedial part of ARC and exhibit a strong nuclear P-STAT3 staining. In panel **(F)**, a group of NPY neurons that are intensely positive for P-STAT3 after CNTF treatment is encircled by a solid line. ARC, arcuate nucleus; ME, median eminence; 3V third ventricle. **(M,N)** Percentage of GFP-expressing NPY and POMC neurons that are also positive for P-STAT3 in the whole ARC **(M)** and it’s rostral (Bregma from −1.46 to −1.70 mm), intermediate (Bregma from −1.70 to −1.94 mm) and caudal (Bregma from −1.94 to 2.18 mm) subdivisions **(N)** from vehicle-injected (CTRL) and CNTF-treated (CNTF) normally fed mice. All pictures were taken from coronal hypothalamic sections at Bregma −1.70 mm. Data (*n* = 3) are mean ± SEM, *p < 0.05 compared to control mice (unpaired student’s *t*-test).

Since fasting alters the structural organization of the ME and modulates the metabolic signals from circulating substances to ARC neurons (Langlet et al., 2013a), the experiments were also conducted in normally fed mice. Vehicle-injected mice exhibited low to moderate P-STAT3 staining in 36.74% ± 0.63 of GFP-expressing NPY neurons (Figures 2A–C,M) and in 30.88% % ± 1.73 of GFP-expressing POMC neurons (Figures 2G–I,M), which were uniformly distributed in the ARC. Surprisingly, CNTF administration led to a reduction in the number of P-STAT3-positive neurons in both mouse models were, however, the fewer P-STAT3-positive cells exhibited a strong, or very strong, nuclear staining (Figures 2D–F,J–L). Thus, after CNTF treatment strong P-STAT3 nuclear staining was detected in 25.50% ± 1.17 of GFP-expressing NPY neurons and only in 2.84% ± 0.45 of GFP-expressing POMC neurons (Figure 2M). As in fasted animals, also in normally fed animals, the vast majority of CNTF-responsive neurons were NPY neurons located in the ventromedial ARC facing the ME (Figure 2F, solid line circle). Assessment of the distribution of CNTF-responsive neurons in treated animals revealed that P-STAT3-positive NPY neurons progressively increased along with the rostrocaudal extent of ARC, whereas P-STAT3-positive POMC neurons were uniformly distributed in the rostral, intermediate and caudal regions (Figure 2N). Finally, in vehicle-injected animals 50.62% ± 1.71 of P-STAT3 immunoreactive neuronal nuclei were located into the NPY neurons and 24.08% ± 1.58 of them were located into the POMC neurons; after CNTF administration, P-STAT3-positive neuronal nuclei were found into 74.11% ± 1.21 of NPY neurons and into 10.41% ± 0.27 of POMC neurons. Thus, in striking contrast to what found in fasted animals, normally fed control and CNTF-treated mice displayed a substantial number (about 25% in vehicle-injected mice and about 15% in CNTF-treated mice) of P-STAT3 positive neurons that were located neither into NPY nor POMC neurons. These data suggest that in fed animals some CNTF actions on hypothalamic ARC could be mediated by non-NPY and non-POMC neurons.

### CNTF Predominantly Activates the JAK2-STAT3 Pathway in ARC NPY Neurons Located Outside the BBB

**Figure 3:**
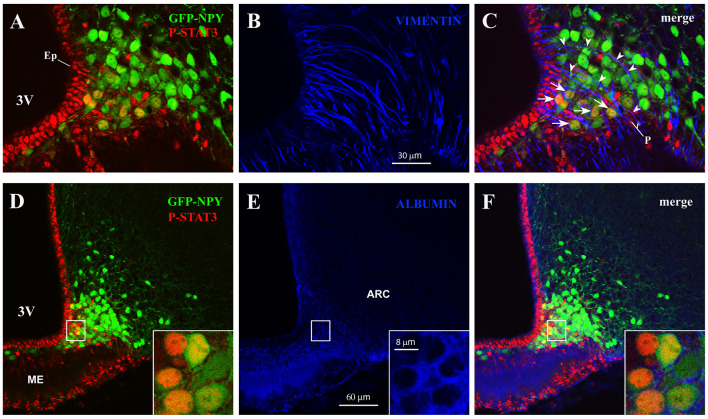
CNTF activates the JAK2-STAT3 pathway in NPY neurons located outside the blood-brain barrier (BBB). **(A–C)** Representative confocal images from a 24-h fasted CNTF-treated GFP-expressing NPY mouse showing clustered P-STAT3-positive ARC NPY neurons **(A)** lying among or close to vimentin-positive β1-tanycyte projections (**B**, blue). **(D–F)** Representative confocal images from a 24-h fasted CNTF-treated GFP-expressing NPY mouse showing that P-STAT3-positive ARC NPY neurons **(D)** are located in an area showing albumin immunoreactivity (**E**, blue). In panel **(E)**, strong albumin staining in the ME progressively declines as it approaches the hypothalamic parenchyma, which is virtually devoid of specific staining. Insets of panels **(D–F)** are high-resolution confocal images from the area framed in panel **(D)**, showing that the soma of some CNTF-responsive NPY neurons is surrounded by albumin immunoreactivity. ME, median eminence; 3V third ventricle; Ep, ependymal layer; P, pial surface. All pictures were taken from coronal hypothalamic sections at Bregma −1.70 mm.

In the ME, β2-tanycytes—which line the floor of the third ventricle—provide an efficient barrier between the ME milieu and the ventricular cerebrospinal fluid (Mullier et al., 2010; Langlet et al., 2013b); in contrast, β1-tanycytes—which line the lateral recesses of the third ventricle and stretch between the ventricular and the pial surface—are believed to form a lateral barrier separating the intercellular space of the ME from that of the ARC (Krisch et al., 1983; Peruzzo et al., 2000). To gain greater insight into the spatial distribution of CNTF-responsive ARC neurons, hypothalamic sections from CNTF-treated GFP-expressing NPY mice were stained with P-STAT3 and vimentin antibodies. The results showed that CNTF-sensitive NPY neurons were often clustered and lay among or close to vimentin-positive β1-tanycyte projections (Figures 3A–C). At this site, a significant number of NPY neurons have been described outside the BBB (Faouzi et al., 2007; Olofsson et al., 2013). Next, to detect neuronal bodies in direct contact with the circulation, we employed immunoreactivity against endogenous albumin, which escapes from fenestrated capillaries but does not penetrate the BBB (Saunders et al., 2015). These experiments showed that in both fasted and normally fed animals virtually all CNTF-responsive NPY neurons were surrounded by albumin (Figures 3D–F). Collectively, these experiments show that exogenous CNTF predominantly activates the JAK2-STAT3 pathway in a distinctive population of ARC NPY neurons which lie outside the BBB and are directly exposed to blood-borne substances.

### CNTF Activates Extracellular Signal-Regulated Kinase (ERK) Signaling in ME Ependymal Cells and β-Tanycytes

**Figure 4:**
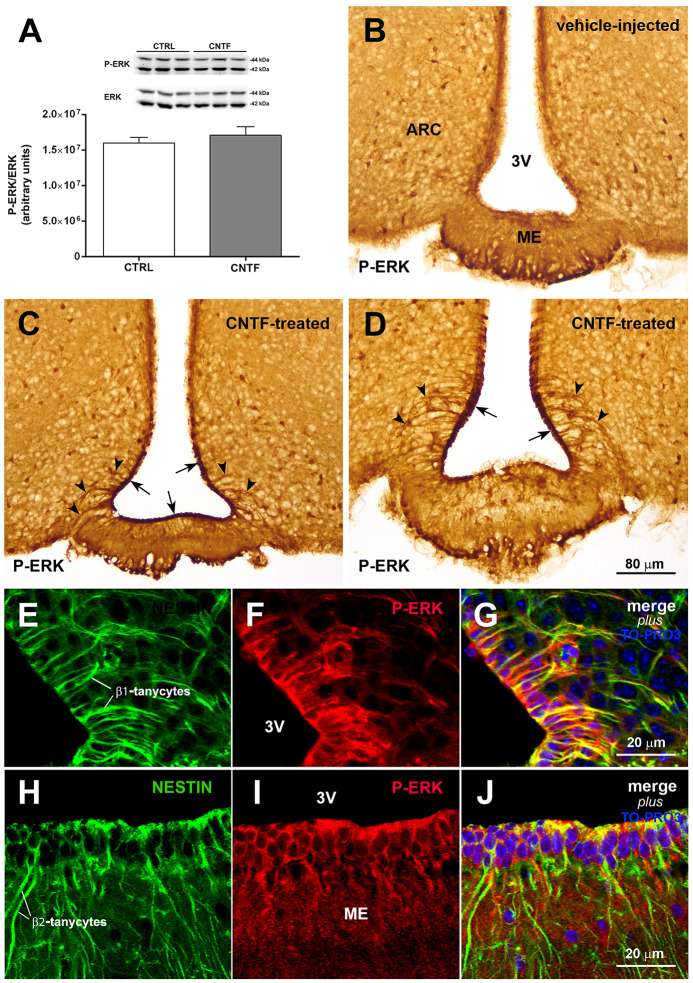
CNTF activates the extracellular signal-regulated kinase (ERK) pathway in ME tanycytes. **(A)** Representative immunoblot and P-ERK quantification in hypothalamic protein extracts from vehicle-injected (CTRL) and CNTF-treated (CNTF) mice. Data (three mice) are mean ± SEM (unpaired student’s *t*-test). **(B–D)** Representative immunoperoxidase images showing P-ERK immunoreactivity in hypothalamic coronal sections from a vehicle-injected **(B)** and a CNTF-treated **(C,D)** mouse. Although P-ERK-positive neuron-like cells are found in the hypothalamic parenchyma in both animals, specific P-ERK staining in the ependymal layer (**C**, arrow) and tanycyte-like projections (**D**, arrowheads) are detected only in the CNTF-treated mouse. **(E–J)** Representative confocal images from a CNTF-treated mouse showing numerous nestin-positive β1-(**E**, green) and β2-tanycytes (**H**, green) that also express P-ERK immunoreactivity (**F,I**, red). ARC, arcuate nucleus; ME, median eminence; 3V third ventricle. All pictures were taken from coronal hypothalamic sections at Bregma −1.70 mm, except for the picture in 4D that was taken from a coronal hypothalamic section at Bregma −2.06 mm.

Induction of extracellular signal-regulated kinase (ERK) signaling in β-tanycytes has been associated with the promotion of leptin transport into the cerebrospinal fluid of the third ventricle, from which leptin diffuses through the hypothalamus (Balland et al., 2014). To explore a possible role for CNTF in this process, the possible activation of ERK signaling was evaluated in the hypothalamus of normal mice subjected to CNTF treatment. Western blotting documented no significant differences in the hypothalamic expression of phospho-ERK (P-ERK) between treated and control mice (Figure 4A). By immunohistochemistry, P-ERK staining was detected in neuron-like cells distributed in several nuclei of the tuberal hypothalamus, including the ARC, in both control and CNTF-treated mice (Figures 4B–D; Morikawa et al., 2004). Even whether a detailed quantitative analysis was not performed, the intensity of this staining and the number of P-ERK-positive neurons did not show visually evident differences between the two conditions. However, unlike control mice (Figure 4B) treated mice showed strong P-ERK immunoreactivity in the ependyma lining the infundibular recess of the third ventricle (Figures 4C,D, arrows). In these mice, tanycyte-like cells exhibited long and strongly stained processes extending from the ependyma into adjacent tissue (Figures 4C,D, arrowheads). Double immunofluorescence and confocal microscopy analyses using the tanycyte marker nestin confirmed that several CNTF-induced P-ERK-positive ependymal cells were β1- (Figures 4E–G) and β2-tanycytes (Figures 4H–J).

### CNTF Promotes Leptin’s Entry Into and Action on the Hypothalamus of Normal-Weight Mice

**Figure 5:**
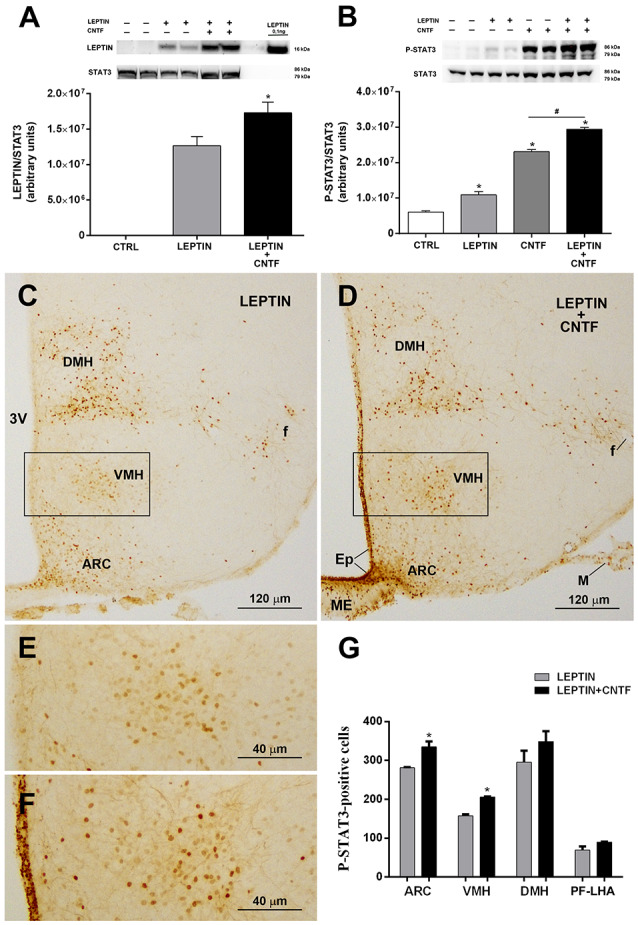
CNTF administration promotes leptin’s entry and STAT3 phosphorylation in the hypothalamus of normal mice. **(A)** Representative immunoblot and quantification of leptin content in hypothalamic protein extracts from vehicle-injected (CTRL), leptin- and leptin/CNTF-treated mice. Data (three mice) are mean ± SEM, *p < 0.05 compared to leptin-injected mice (unpaired student’s *t*-test). **(B)** Representative immunoblot and quantification of P-STAT3 content in hypothalamic protein extracts from vehicle-injected (CTRL), leptin-, CNTF- and leptin/CNTF-treated mice. Data (three mice) are mean ± SEM, *p < 0.05 compared to vehicle-injected mice, ^#^*p* < 0.05 compared to CNTF-treated mice (one-way ANOVA). **(C–F)** Representative P-STAT3 immunoperoxidase staining from a leptin-treated **(C,E)** and a leptin/CNTF-treated **(D,F)** mouse. **(E,F)** Are enlargements of the areas framed in **(C,D)**, respectively. ARC, arcuate nucleus; VMH, ventromedial hypothalamus; DMH, dorsomedial hypothalamic nucleus; f, fornix; Ep, ependyma; ME, median eminence; M, meninges. **(G)** Number of P-STAT3-positive neurons in different hypothalamic nuclei in leptin- and leptin/CNTF-treated mice. PF-LHA, perifornical-lateral hypothalamic area. All pictures were taken from coronal hypothalamic sections at Bregma −1.70 mm. Data (three mice) are mean ± SEM, *p < 0.05 compared to leptin-treated mice (unpaired student’s *t*-test).

The results described above suggest that by activating ERK signaling in β-tanycytes exogenous CNTF could promote leptin’s entry into the hypothalamus. To test this hypothesis, leptin was quantified by Western blotting in whole hypothalamic protein extracts from control mice and mice treated with leptin or leptin/CNTF. Leptin was undetectable in control mice, whereas it was readily detectable in leptin- and leptin/CNTF-treated mice. Interestingly, a slightly but significantly higher leptin content was found in hypothalamic protein extracts from mice treated with leptin/CNTF than in leptin-treated mice (Figure 5A). To establish whether CNTF administration was associated with increased leptin signaling, we first evaluated by Western blotting the expression of P-STAT3 in the hypothalamus of control mice and mice treated with leptin, CNTF or leptin/CNTF. Compared to control mice, leptin-induced a significant increase in STAT3 phosphorylation; CNTF induced a strong increase in P-STAT3, and their co-administration increased STAT3 phosphorylation to a significantly greater extent than CNTF alone (Figure 5B). As expected, the immunohistochemical expression and distribution of P-STAT3-positive neurons in the hypothalamic parenchyma of mice treated with leptin or leptin/CNTF showed that in the former mice JAK2-STAT3 signaling was activated in several neurons of the ARC, VMH, DMH, and PF-LHA, whereas in leptin/CNTF mice P-STAT3 immunoreactivity was strong in ME cells and the ependyma in addition to these sites. However, a comparison of corresponding sections processed in the same immunohistochemical experiment in standardized conditions disclosed that, compared to leptin-treated mice (Figure 5C), P-STAT3 immunoreactivity in leptin/CNTF-treated mice was stronger and/or involved a larger number of hypothalamic neurons (Figure 5D). In particular, the VMH contained more intensely stained and more numerous P-STAT3-positive neurons in leptin/CNTF-treated mice (Figure 5F) than in leptin-treated mice (Figure 5E). Morphometric analysis showed that leptin/CNTF treatment was associated with an increased number of P-STAT3-positive neurons in all hypothalamic nuclei targeted by leptin, but that it achieved significance only in the ARC and VMH (Figure 5G). Notably, in obese *db*/*db* mice lacking the leptin receptor, immunohistochemistry showed that CNTF administration activated the ERK pathway in ME ependymal cells and tanycytes, as in normal mice (Supplementary Figure S1A). However, when compared with CNTF-treated *db*/*db* mice, leptin/CNTF-treated *db*/*db* mice did not exhibit any increase of STAT3 phosphorylation in hypothalamic protein extracts (Supplementary Figure S1B). Additionally, in both groups of animals P-STAT3 immunoreactivity was detected in the ependymal layer and some ARC neurons (Supplementary Figures S1C,D), being such staining due to CNTF administration (see previous results). However, we did not found any appearance of P-STAT3-positive neurons in hypothalamic feeding centers, including the VMH, the DMH, and the PF-LHA, and the number of P-STAT3-positive ARC neurons did not change between the CNTF- and leptin/CNTF-treated mice (Supplementary Figure S1E). Collectively, these data show that in normal mice CNTF administration enhances leptin’s entry into the brain and the hypothalamic responsiveness to leptin in LepRb-bearing neurons.

### CNTF Promotes Leptin’s Entry Into and Action on the Hypothalamus of Obese Mice Fed an HFD

**Figure 6:**
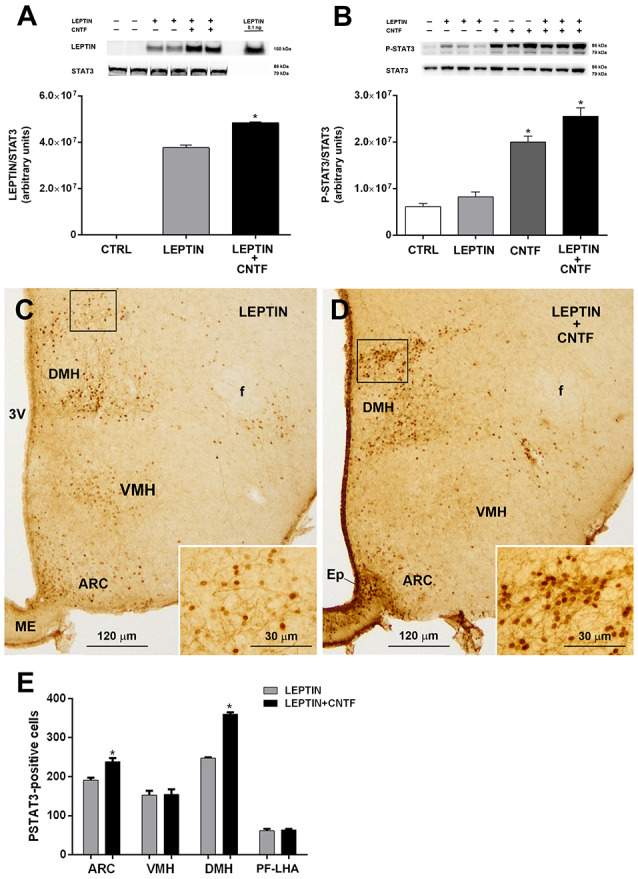
CNTF administration promotes leptin’s entry and STAT3 phosphorylation in the hypothalamus of high-fat diet (HFD) obese mice. **(A)** Representative immunoblot and quantification of leptin content in hypothalamic protein extracts from vehicle-injected (CTRL), leptin- and leptin/CNTF-treated mice. Data (three mice) are mean ± SEM, *p < 0.05 compared to leptin-injected mice (unpaired student’s *t*-test). **(B)** Representative immunoblot and quantification of P-STAT3 content in hypothalamic protein extracts from vehicle-injected (CTRL), leptin-, CNTF- and leptin/CNTF-treated mice. Data (three mice) are mean ± SEM, *p < 0.05 compared to vehicle-injected mice (one-way ANOVA). **(C,D)** Representative P-STAT3 immunoperoxidase staining from a leptin-treated **(C)** and a leptin/CNTF-treated **(D)** mouse. Insets of **(C,D)** are enlargements of the corresponding framed areas. ARC, arcuate nucleus; VMH, ventromedial hypothalamus; DMH, dorsomedial hypothalamic nucleus; f, fornix; Ep, ependyma; ME, median eminence. **(E)** Number of P-STAT3-positive neurons in different hypothalamic nuclei in leptin- and leptin/CNTF-treated mice. PF-LHA, perifornical-lateral hypothalamic area. All pictures were taken from coronal hypothalamic sections at Bregma −1.70 mm. Data (three mice) are mean ± SEM, *p < 0.05 compared to leptin-treated mice (unpaired student’s *t*-test).

Molecules that promote leptin’s entry into the brain and leptin signaling are very interesting for therapeutic purposes, since most obese subjects are leptin-resistant, with high circulating leptin levels that do not reduce appetite or increase energy expenditure, as they do in healthy subjects (Maffei et al., 1995; Woods et al., 2000). To determine whether CNTF promotes leptin’s entry and action also in the hypothalamus of leptin-resistant mice, its effects were evaluated in mice rendered obese by a 10-week HFD (Supplementary Figures S2A,B). Compared to normally fed animals, vehicle-injected HFD mice showed a lower P-ERK content in hypothalamic protein extracts (Supplementary Figure S2C) that was paralleled by a strong reduction, or even disappearance, of P-ERK immunoreactivity in hypothalamic neurons (not shown). In HFD mice, CNTF administration induced a significantly increased expression of hypothalamic P-ERK in Western blots (Supplementary Figure S2C) and P-ERK immunoreactivity in ME ependymal cells and tanycytes (Supplementary Figures S2D–G). As in normally fed mice, leptin levels were significantly higher in the hypothalamus of leptin/CNTF-treated HFD animals than in leptin-treated HFD mice (Figure 6A). The Western blots also showed that leptin increased STAT3 phosphorylation in the hypothalamus of obese mice, though not to a significant extent, likely due to HFD-induced leptin resistance; that CNTF induced strong JAK2-STAT3 signaling; and that their co-administration resulted in an even greater increase in P-STAT3 expression (Figure 6B) that was not however significant compared to CNTF alone. As regards the immunohistochemical experiments, a comparison of corresponding coronal sections from leptin- (Figure 6C) and leptin/CNTF-treated mice (Figure 6D) showed that in the latter animals P-STAT3 immunoreactivity was again stronger and/or present in a larger number of neurons. In some nuclei, especially the DMH (insets of Figures 6C,D), co-treatment also induced more numerous and more intensely stained neural fibers. Morphometric analysis showed that co-treatment induced a significant increase in P-STAT3-positive neurons in the ARC and the DMH (Figure 6E). Collectively, these data show that CNTF retains the ability to promote leptin entry and signaling in hypothalamic feeding centers even in obese mice fed an HFD.

## Discussion

In experimental animals and humans, systemic CNTF treatment induces rapid and sustained weight loss as a result of reduced food intake (Lambert et al., 2001; Anderson et al., 2003; Ettinger et al., 2003; Janoschek et al., 2006). Its ability to reduce food intake has largely been ascribed to activation of the JAK2-STAT3 pathway in hypothalamic ARC neurons (Lambert et al., 2001; Anderson et al., 2003; Janoschek et al., 2006). In this study, assessment of P-STAT3 immunoreactivity in the hypothalamus of GFP-expressing NPY and GFP-expressing POMC mice treated by acute intraperitoneal administration of recombinant mouse CNTF provided clear evidence that CNTF truly and exclusively acts on ARC neurons and a few scattered ME neurons. However, our data also highlight novel aspects of the hypothalamic action of CNTF. First, exogenous CNTF mainly acts on NPY neurons and does not seem to exert a marked effect on the anorexigenic POMC neurons, most of which lie scattered in the laterodorsal hypothalamic ARC; notably, in normally fed mice and mice fasted for 24 h, only a very limited percentage of POMC neurons located in the ventromedial hypothalamic ARC appeared to respond to CNTF. These findings agree with previous reports that the anorectic effect of CNTF is maintained in melanocortin-4 receptor-deficient mice, where the anorexigenic neural pathways downstream the POMC neurons are impaired (Marsh et al., 1999). Also, the present results show that in normally fed mice and fasted mice, exogenous CNTF strongly activated the JAK2-STAT3 pathway in a population of NPY neurons that are distinctively located in the most ventromedial part of the ARC on the border with the ME. These CNTF-responsive NPY neurons often formed clusters and lay among the projections of β1-tanycytes. The cell body of β1-tanycytes is found in the ependyma lining the lateral sides of the infundibular recess, and long branched projections emerging from the cell body reach the ventral pial surface. In this way, β1-tanycytes are believed to form a lateral barrier preventing the diffusion of blood-borne substances extravasated from the fenestrated portal capillaries of the ME to the adjacent ARC (Krisch et al., 1983; Peruzzo et al., 2000). The anatomical position of the vast majority of the CNTF-responsive NPY neurons closely resembles the position of the recently characterized NPY neurons that lie outside the BBB (Olofsson et al., 2013; Yulyaningsih et al., 2017). These neurons have been suggested to sense the smallest changes in plasma metabolic signals and to serve as fast first-line responders to metabolically relevant circulating hormones and peptides (Yulyaningsih et al., 2017). Collectively, these data suggest that circulating CNTF crosses the fenestrated capillaries of the ME, diffuses over the ME milieu, and targets the ARC NPY neurons located outside the BBB, where it activates JAK2-STAT3 signaling and rapidly induces a satiety effect.

However, CNTF diffusion over the ME also involves the stimulation of several non-neuronal cells. Accordingly, a substantial number of CNTF-responsive glial cells expressing markers of immaturity, including nestin and vimentin (Severi et al., 2012, 2015), could be targeted by CNTF and give rise to neuronal cells (Kokoeva et al., 2005; Lee et al., 2012). Interestingly, in this study, we also found that CNTF distinctively activates ERK signaling in β-tanycytes and in the ependymal cells lining the ME, an effect that has been linked to increased leptin transport to the hypothalamic feeding centers (Balland et al., 2014). According to this model, LepR-expressing β-tanycytes bind circulating leptin extravasated from the fenestrated capillaries of the ME, the LepR-leptin complex is transported by transcytosis along β-tanycytes, and leptin is secreted into the lumen of the third ventricle; from here it crosses the ependyma lining the lateral walls of the third ventricle and finally diffuses over the hypothalamic parenchyma. However, how circulating leptin gains access to hypothalamic feeding centers is still an open question. Although active transport across the BBB is an attractive hypothesis (Banks et al., 1996; Di Spiezio et al., 2018), the molecular carrier involved has yet to be identified. Since the hypothesis involving ERK-dependent transport by β-tanycyte has been undermined by the failure to detect LepR mRNA in hypothalamic tanycytes (Yoo et al., 2019), LepR-independent mechanisms should be considered to explain the carrier role of tanycytes. Finally, high-resolution imaging techniques have recently suggested that the choroid plexus (Harrison et al., 2019), which expresses high levels of LepR (Bjørbaek et al., 1998) and, interestingly, also of CNTFR (Kelly et al., 2004; Severi et al., 2012), maybe an important route of leptin entry into the brain. The increased amount of leptin, found in our study, in the hypothalamus of normally fed and HFD mice treated with leptin/CNTF and the increased P-STAT3 immunoreactivity detected in the ARC and VMH of normally fed leptin/CNTF mice and the ARC and DMH of leptin/CNTF HFD obese mice concerning the leptin-treated mice suggest that CNTF co-administered with leptin can to some extent promote leptin’s entry into and action on the mouse hypothalamus. Why CNTF/leptin co-administration increases the percentage of P-STAT3-positive neurons in the VMH of normal-weight mice and the DMH of HFD obese mice remains unknown and deserves further studies. This may depend on the kinetics of leptin spreading through the hypothalamic parenchyma which may be different in normal and obese mice; however, any role of CNTF in this process can only be assessed in future studies by applying different doses of recombinant CNTF and different time points of treatments. Interestingly, in a previous study of HFD mice, a common model of obesity characterized by high circulating leptin and leptin resistance, CNTF administration induced P-STAT3 immunoreactivity in ME cells, the ependyma and ARC neurons but also, to a lesser and variable extent, in some neurons located in the DMH, VMH, PF-LHA and the mammillary body (Severi et al., 2013). In the light of the present data, this neuronal staining could be ascribed to a CNTF-dependent increased leptin entry into and action on the hypothalamus of HFD obese mice. Notably, such staining has been described neither in CNTF-treated *ob*/*ob* mice genetically lacking leptin (Severi et al., 2013) nor in leptin/CNTF-treated *db*/*db* mice genetically lacking LepR (current results).

Accordingly, we feel that the present findings and earlier data suggest that circulating CNTF induces a reduction in food intake through multiple and redundant mechanisms that are probably characterized by different kinetics: rapid inhibition of orexigenic NPY neurons located in the ARC (outside the BBB), consequent promotion of leptin entry into the brain and, possibly, generation of new leptin-responsive neurons in the mediobasal hypothalamus. Obesity is a steadily spreading disease that is associated with a heightened risk of developing a variety of severe medical conditions including insulin resistance and type 2 diabetes, dyslipidemia, non-alcoholic fatty liver, cardiovascular disease, and even some cancers. Common obesity is associated with leptin resistance, a major aspect of the pathophysiology of this disease (Maffei et al., 1995; Woods et al., 2000). Even though the underlying molecular mechanisms are not yet entirely clear, impaired leptin transport into the brain is viewed as an important contributor to leptin resistance (Banks et al., 1999; El-Haschimi et al., 2000). Our data, showing that exogenously administered CNTF acts on NPY neurons located outside the BBB and promotes leptin’s entry into the brain, may explain its effectiveness in leptin-resistant obese patients (Ettinger et al., 2003) and animals (Lambert et al., 2001). Investigation of the multiple, redundant mechanisms by which CNTF induces a reduction in food intake has the potential to suggest novel treatments for morbid obesity.

Viewed from a different perspective, our data are also consistent with a possible metabolic role for endogenous CNTF, still a largely unexplored topic. CNTF is a member of the interleukin-6 cytokine family, which includes molecules that are quickly induced by a wide range of pathological stimuli and rapidly secreted into the circulation. The few available studies show that CNTF is barely detectable in the blood of healthy subjects, whereas its concentration increases in patients with conditions as diverse as amyotrophic lateral sclerosis (Laaksovirta et al., 2008), septic shock (Guillet et al., 1995) and autism (Brondino et al., 2018). Recently, a central CNTF-dependent tanycyte pathway has been suggested to be involved in stress-induced cortical alertness and, possibly, in the pathogenesis of post-traumatic stress disorder (Alpár et al., 2018). Altogether, these findings suggest that endogenous circulating CNTF could be a novel metabolic regulator acting on the tuberal hypothalamus to match food intake with distinctive somatic and behavioral pathological conditions.

## Data Availability Statement

All datasets generated for this study are included in the article/Supplementary Material.

## Ethics Statement

The animal study was reviewed and approved according to Council Directive 2010/63/UE and all experiments were approved by the Italian Health Ministry (Authorization No. 405/2018-PR).

## Author Contributions

WV and IS: performance of experiments, data analysis, interpretation, and manuscript writing. JP and EDM: performance of experiments, data analysis, and interpretation. MMai and MMaf: providing reagents and critical revision. SC: critical revision of the manuscript and financial support. AG: conception and design, financial support, data analysis and interpretation, manuscript writing, and final approval of the manuscript.

## Conflict of Interest

The authors declare that the research was conducted in the absence of any commercial or financial relationships that could be construed as a potential conflict of interest.
