# Supplementary material for: Ciliary Neurotrophic Factor Acts on Distinctive Hypothalamic Arcuate Neurons and Promotes Leptin Entry Into and Action on the Mouse Hypothalamus
Source: Front Cell Neurosci. 2020 May 21;14:140. doi: 10.3389/fncel.2020.00140 (PMC7253709; doi:10.3389/fncel.2020.00140)
Supplement: Supplementary file 1 [file Data_Sheet_1.pdf]

Supplementary table 1 – Primary and secondary antibodies used in the study.

TABLE 1A| Primary antibodies.

Antibodies	Host*	Diluition	Source
**Albumin**	G	1:4000 (IF)	Novus Biologicals, NB600-41532
**ERK1/2**	R	1:1000 (WB)	Cell Signaling Technology, 9102
**GFP**	R	1:300 (IF)	Cell Signaling Technology, 2956
**Leptin**	R	1:1000 (WB)	Peprotech, 500-P68
**Nestin**	M	1:400 (IF)	Merck, MAB353
**pERK1/2 (Thr202/Tyr204)**	R	1:2000 (WB), 1:3000 (IHC, IF)	Cell Signaling Technology, 4370
**pSTAT3 (Tyr705)**	R	1:1000 (WB), 1: 900 (IHC, IF)	Cell Signaling Technology, 9145
**pSTAT3 (Tyr705)**	G	1:600 (IF)	Santa Cruz Biotechnology, sc-7993
**STAT3**	M	1:1000 (WB)	Cell Signaling Technology, 9132
**Vimentin**	G	1:400 (IF)	Santa Cruz Biotechnology, sc-7557

* R, rabbit; M, mouse; G, goat; WB, western blotting; IHC, immunohistochemistry; IF, immunofluorescence.

TABLE 1B| Secondary antibodies.

Conjugated to	React*	Dilution	Source
Fluorophore	R	1:400 (IF)	Invitrogen, A21206
Fluorophore	R	1:400 (IF)	Invitrogen, A31572
Fluorophore	G	1:400 (IF)	Invitrogen, A11055
Fluorophore	G	1:400 (IF)	Invitrogen, A21432
Fluorophore	G	1:400 (IF)	Invitrogen, A21082
Fluorophore	M	1:400 (IF)	Invitrogen, A21202
Peroxidase	R	1:200 (IHC)	Vector Laboratories, BA-1000
Peroxidase	M	1:5000 (WB)	Jackson ImmunoResearch, 715-036-150
Peroxidase	R	1:5000 (WB)	Jackson ImmunoResearch, 711-036-152

* R, rabbit; G, goat; M, mouse.

Supplementary figure 1

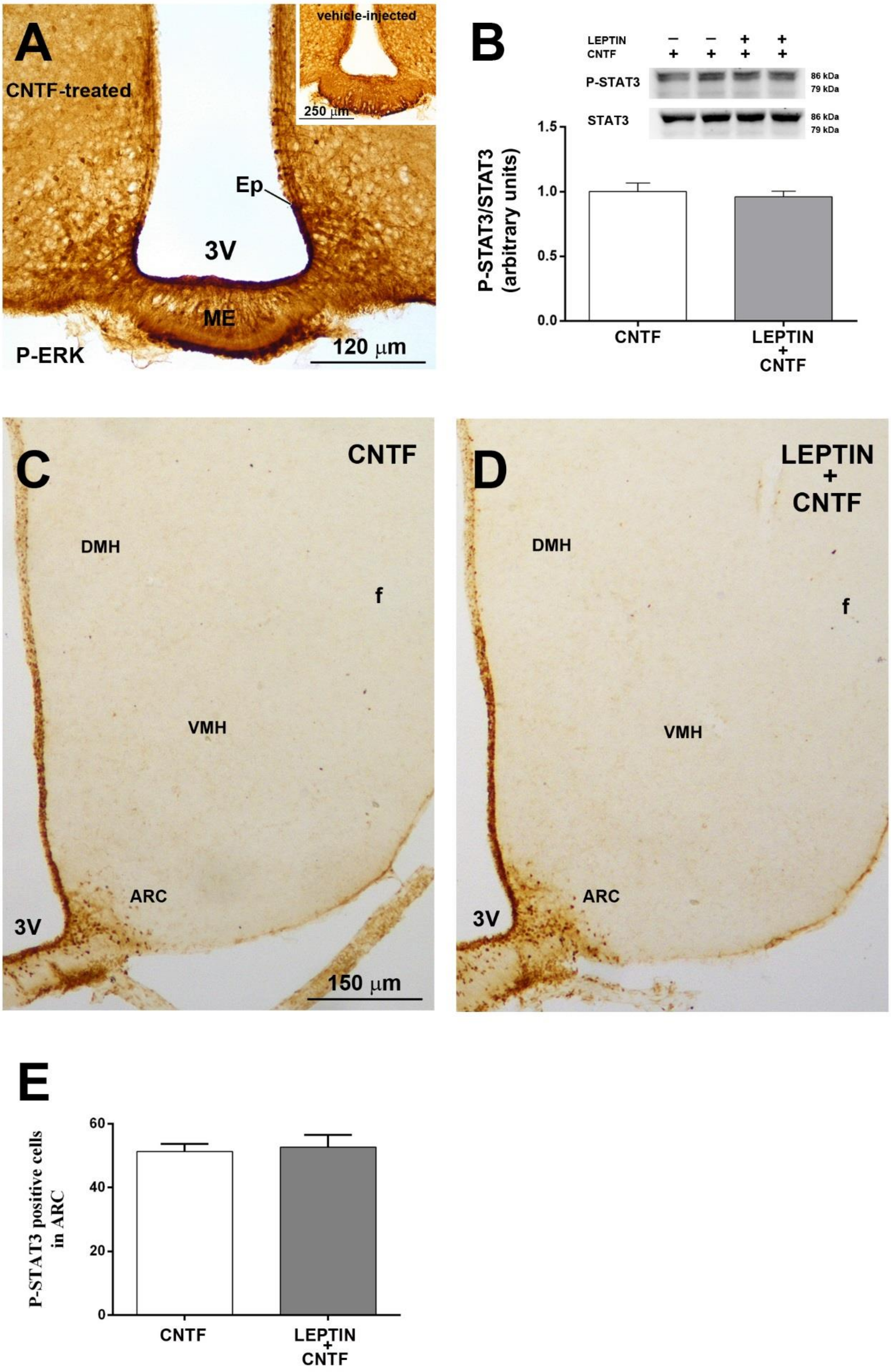

Supplementary figure 1 - CNTF administration to obese *db*/*db* mice. A: Representative immunoperoxidase image showing P-ERK immunoreactivity in the ependyma (Ep) lining the infundibular recess of the third ventricle (3V) from a *db*/*db* mouse treated with CNTF. The inset shows the corresponding region from a vehicle-injected mouse. B: Representative immunoblot and quantification of P-STAT3 content in hypothalamic protein extracts from *db*/*db* mice treated with CNTF or CNTF/leptin. Data (3 mice) are mean ± SEM (one-way ANOVA). C and D: Representative P-STAT3 immunoperoxidase staining from *db*/*db* mice treated with CNTF (C) or CNTF/leptin (D). ARC, arcuate nucleus; VMH, ventromedial hypothalamus; DMH, dorsomedial hypothalamic nucleus; f, fornix; All pics were taken from coronal hypothalamic sections at bregma – 1.70 mm. E: Number of P-STAT3-positive neurons in the ARC of CNTF- and CNTF/leptin-treated mice from three coronal sections. Data (3 mice) are mean ± SEM (unpaired Student's t-test).

Supplementary figure 2

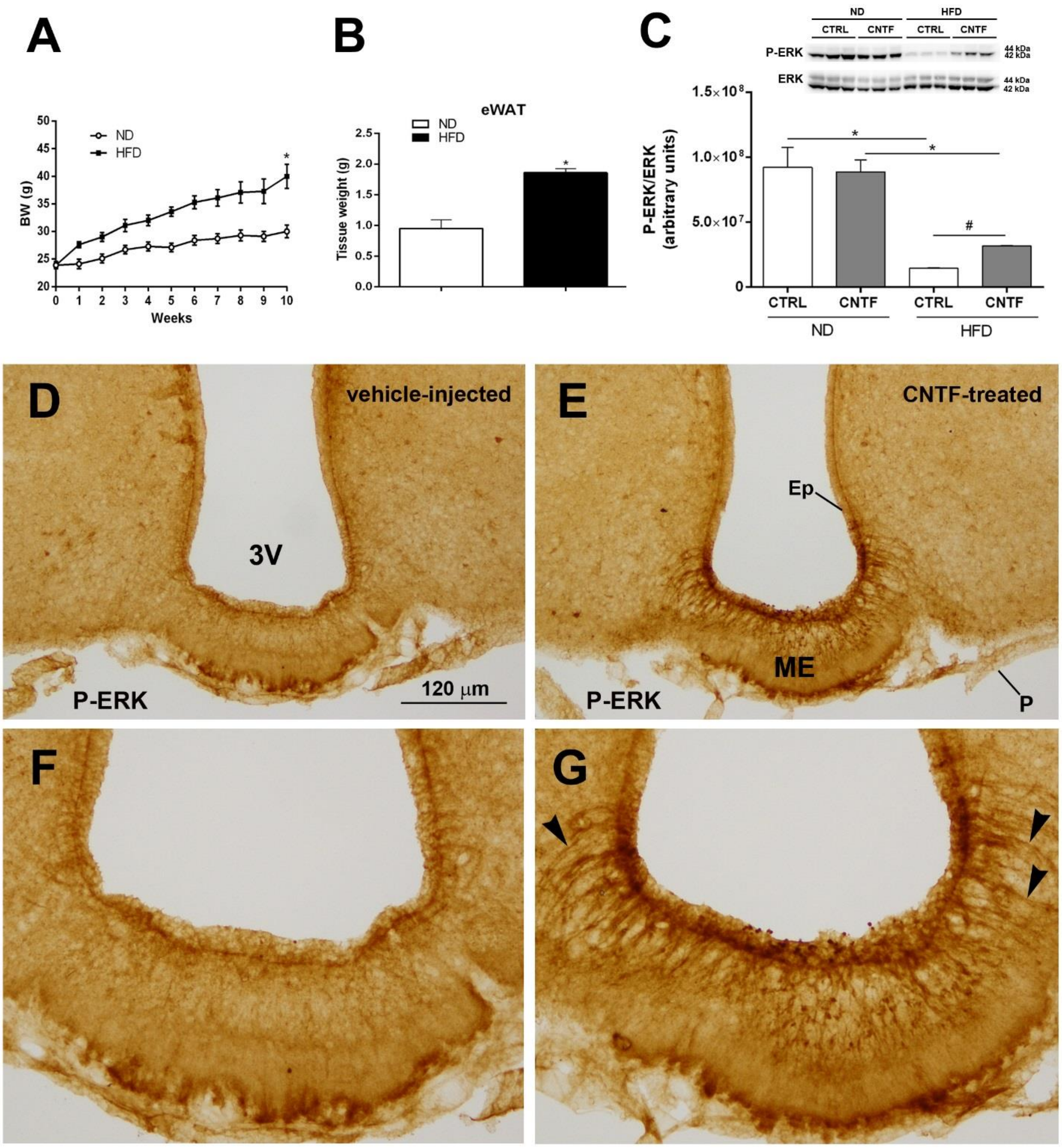

Supplementary figure 2 – Activation of ERK signaling by CNTF in HFD obese mice. A: Body weight of mice fed a normal diet (ND) and a high fat diet (HFD) for 10 weeks. Data (3 mice) are mean ± SEM, (unpaired Student's t-test), *p<0.05 comparison between ND and HFD mice. B: Tissue weight of the epididymal fat depots from ND and HFD mice. Data (3 mice) are mean ± SEM, (unpaired Student's t-test), *p<0.05 comparison between ND and HFD mice. C: Representative immunoblot and P-ERK quantification in hypothalamic protein extracts from vehicle-injected (CTRL-ND) and CNTF-treated (CNTF-ND) mice fed a normal diet (ND) and vehicle-injected (CTRL-HFD) and CNTF-treated (CNTF-HFD) mice fed a HFD. Data (3 mice) are mean ± SEM, *p<0.05 comparison

between ND and HFD mice, #p<0.05 comparison between CNTF-treated and vehicle-injected HFD mice (one-way ANOVA). D-G: Representative immunoperoxidase images showing P-ERK immunoreactivity in a vehicle-injected HFD mouse (D and F) and in a CNTF-treated HFD mouse (E and G). In this latter, arrowheads indicate tanycyte-like projections that are strongly positive for P-ERK. F and G are enlargements of the median eminence (ME) of D and E, respectively. 3V, third ventricle; Ep, ependymal layer, P, pial surface. All pics were taken from coronal hypothalamic sections at bregma – 1.70 mm.
